# 75 Patient Reported Outcome Measures Associated with Burn Severity

**DOI:** 10.1093/jbcr/irac012.078

**Published:** 2022-03-23

**Authors:** Golddy M Saldana, David Greenhalgh, Soman Sen, Tina L Palmieri, Kathleen S Skipton Romanowski

**Affiliations:** UC Davis School of Medicine, Sacramento, California; ; UC Davis, Sacramento, California; UC Davis and Shriners Hospitals for Children Northern California, Sacramento, California; UC Davis, Sacramento, California

## Abstract

**Introduction:**

Burns continue to be a leading cause of unintentional injury sustained by children. The lasting effects of burn treatment on pediatric patients’ quality of life are still poorly understood. Patient-Reported Outcomes Measurement Information System (PROMIS) is a reliable tool that evaluates a patient’s physical, mental, and social health. To measure the impact of burn injuries on quality of life, we integrated the use of PROMIS into our outpatient clinic. Our aim for this study is to determine the effects of patient demographics, burn treatment, and burn characteristics on PROMIS scores.

**Methods:**

A retrospective review of patient and PROMIS data collected at our institution from June 2016 to August 2019 was conducted. Patient demographics, burn injury characteristics, PROMIS Pediatric Mobility, Upper Extremity, Pain Interference, and Peer Relationships scores were collected. Statistical analysis was conducted using Wilcoxon Two-Sample Test, Chi-Square, and Pearson’s Correlation Coefficient.

**Results:**

163 pediatric burn patients completed PROMIS questionnaires. The median injury age was 5.94 years (interquartile range (IQR)= 6.9) and had a median total body surface area of 15% (IQR = 25.5). Individual burn characteristics did not impact Peer Relationships scores; however, they did have a significant effect on Upper Extremity, Mobility, and to a lesser extent, Pain Interference scores. Older injury age (-0.24, p = 0.02), longer length of stay (-0.25, p = 0.02), larger total body surface area (-0.27, p = 0.009), and more operating room visits (-0.28, p = 0.006), were associated with worse Upper Extremity scores. Similarly, older injury age (-0.2, p = 0.04), longer length of stay (-0.25, p = 0.01), more days in the intensive care unit (-0.24, p = 0.01), and more operating room visits (-0.26, p = 0.01) impacted Mobility scores. Worse Pain Interference scores were correlated with longer length of stay (0.24, p=0.01), and larger total body surface area (0.19, p=0.05). Upper Extremity scores correlated with Mobility scores (0.60, p < 0.0001); Pain Interference (-0.23, p < 0.02) and Upper Extremity (0.3, p < 0.003) scores correlated with Peer Relationships scores; and Upper Extremity (-0.48, p < 0.0001) and Mobility (-0.42, p < 0.0001) scores correlated with Pain Interference scores.

**Conclusions:**

Peer Relationships scores are not influenced by patient demographics or burn characteristics. This is in contrast with Upper Extremity, Mobility, and Pain Interference scores which are all directly affected by burn characteristics. The patients’ impression of their upper extremity function has the greatest impact on their perceived pain, peer relationships, and mobility.

